# Arbuscular Mycorrhiza Alleviates Restrictions to Substrate Water Flow and Delays Transpiration Limitation to Stronger Drought in Tomato

**DOI:** 10.3389/fpls.2018.00154

**Published:** 2018-02-16

**Authors:** Michael Bitterlich, Martin Sandmann, Jan Graefe

**Affiliations:** Leibniz-Institute of Vegetable and Ornamental Crops, Großbeeren, Germany

**Keywords:** arbuscular mycorrhiza, water retention, drought, tomato, transpiration, soil properties, hydraulic conductivity, root water uptake

## Abstract

Arbuscular mycorrhizal fungi (AMF) proliferate in soil pores, on the surface of soil particles and affect soil structure. Although modifications in substrate moisture retention depend on structure and could influence plant water extraction, mycorrhizal impacts on water retention and hydraulic conductivity were rarely quantified. Hence, we asked whether inoculation with AMF affects substrate water retention, water transport properties and at which drought intensity those factors become limiting for plant transpiration. *Solanum lycopersicum* plants were set up in the glasshouse, inoculated or not with *Funneliformis mosseae*, and grown for 35 days under ample water supply. After mycorrhizal establishment, we harvested three sets of plants, one before (36 days after inoculation) and the second (day 42) and third (day 47) within a sequential drying episode. Sampling cores were introduced into pots before planting. After harvest, moisture retention and substrate conductivity properties were assessed and water retention and hydraulic conductivity models were fitted. A root water uptake model was adopted in order to identify the critical substrate moisture that induces soil derived transpiration limitation. Neither substrate porosity nor saturated water contents were affected by inoculation, but both declined after substrates dried. Drying also caused a decline in pot water capacity and hydraulic conductivity. Plant available water contents under wet (pF 1.8–4.2) and dry (pF 2.5–4.2) conditions increased in mycorrhizal substrates and were conserved after drying. Substrate hydraulic conductivity was higher in mycorrhizal pots before and during drought exposure. After withholding water from pots, higher substrate drying rates and lower substrate water potentials were found in mycorrhizal substrates. Mycorrhiza neither affected leaf area nor root weight or length. Consistently with higher substrate drying rates, AMF restored the plant hydraulic status, and increased plant transpiration when soil moisture declined. The water potential at the root surface and the resistance to water flow in the rhizosphere were restored in mycorrhizal pots although the bulk substrate dried more. Finally, substrates colonized by AMF can be more desiccated before substrate water flux quantitatively limits transpiration. This is most pronounced under high transpiration demands and complies with a difference of over 1,000 hPa in substrate water potential.

## Introduction

Biostimulants in agri- and horticulture are defined as substances or microorganisms applied to plants in minute quantities aiming to improve crop quality traits, stress tolerance and nutrient efficiency, without being mineral nutrients, soil improvers or pesticides, which are applied in high quantities (du Jardin, 2015). Those involve humic acids, protein hydrolysates, seaweed extracts, biopolymers (Colla et al., 2015; du Jardin, 2015) and beneficial microbes such as plant growth promoting rhizobacteria (Ruzzi and Aroca, 2015) and arbuscular mycorrhizal fungi (AMF) (Rouphael et al., 2015). AMF in particular are considered as potential biostimulants, because they are able to colonize many important crop species and contribute to the abiotic stress tolerance, disease resistance and nutrient acquisition of their hosts (Rouphael et al., 2015). The provision of phosphorus (P) from soil to plants by AMF is well documented and is based on the increased surface area for P absorption provided by hyphae that proliferate in substrates beyond relatively short ranged root P depletion zones (Smith and Read, 2008). When extraradical hyphae spread in soils or substrates, they will penetrate areas beyond the ambit of roots and compete for resources and space with roots and other microbes. Soils are self-organizing systems, their structure builds up hierarchically and AMF are important contributors (Tisdall and Oades, 1982; Milleret et al., 2009; Daynes et al., 2013). Filamentous AMF hyphae are seen as sticky-string bags that influence physical and chemical properties of the substrate area around them by exudation, enmeshment and entanglement of particles and, the release of intrahyphal components during turnover (Miller and Jastrow, 2000). They have physical contact with roots, have direct access to plant derived carbon and constitute a network, which redistributes organic carbon in the soil (Miller and Jastrow, 2000). In those ways, AMF can contribute positively to the formation and stabilization of aggregation in soils (Augé et al., 2001; Rillig et al., 2002; Piotrowski et al., 2004; Rillig and Mummey, 2006), can affect soil water repellency (Rillig et al., 2010) and logically would affect pore volume, pore distribution and wettability. Similar to roots (Bodner et al., 2014), AMF influence soil structure, which has been investigated extensively, but quantification of the impact of mycorrhizal effects on soil hydraulic properties remains less clear and has gained surprisingly little attention (Querejeta, 2017). Vice versa, an understanding of soil properties is crucial for the efficient use of AMF in biological systems (Frey and Ellis, 1997).

Substrate water retention characteristics and hydraulic conductivity are specific for every soil or substrate and depend on texture (particle size distribution) and structure (particle arrangement) (Querejeta, 2017). The latter could be affected by AMF and in turn, AMF could affect substrate water retention (Augé et al., 2001) and hydraulic conductivity. If influenced by AMF, changes in water retention and hydraulic conductivity could have significant impacts on the ability of the host to extract water and the stress plants would experience at particular levels of substrate moisture. Under moisture stress, AMF may confer drought tolerance to hosts (Ruiz-Lozano et al., 1995; Augé, 2001; Augé et al., 2015). Under defined drought conditions, colonized plants often grow better than their non-mycorrhizal counterparts (e.g., Ruiz-Lozano et al., 1995), stomatal conductance is often enhanced under drought (Khalvati et al., 2005; Augé et al., 2015) and higher drying rates were observed in substrates that contain such symbiotic plant-fungus associations (Khalvati et al., 2005; Ruth et al., 2011). Several hypotheses of underlying mechanisms do exist, but it was also shown that non-host mutant plants growing in a mycorrhizal soil can maintain stomata opening, although there is no functional symbiosis (Augé, 2004). This indicates a substrate originated effect and might be related to changes in substrate water retention and transport properties, which could be caused by e.g., formation of soil structure, a higher degree of particle-particle contact and increases and conservation of pore connectivity by proliferation into additional pores. However, up to date, mycorrhizal effects on water retention have been scarcely examined (Augé et al., 2001, 2004; Bearden, 2001; Daynes et al., 2013) and, to our best knowledge, AMF effects on substrate conductivity as a strict physical property are not yet reported.

Roots do affect soil structure (Bodner et al., 2014) and induction of aggregate formation by roots may be dominant over mycorrhizal effects (Hallett et al., 2009), but roots and AMF together can result in largest changes in substrate hydraulic characteristics (Daynes et al., 2013). In the past, scientist have used root free compartments to study mycorrhizal functioning (George et al., 1992; Ruth et al., 2011), but the realistic scenario for the host is a substrate that contains roots and AMF. Especially, when host physiological responses to substrate properties are examined, roots should not be excluded. Thus we chose an experimental design that uses tomato as the host, which is one of the most important vegetable crops worldwide, compatible with many AMF species and, which allows avoidance of large confounding effects by biomass development. To our experience and to that of others, healthy wild type tomato plants frequently lack strong growth responses, although a functional symbiosis was verified by root colonization, nutritional, physiological and/or metabolic reactions (Pozo et al., 1999; Smith et al., 2004; Neumann and George, 2005; Boldt et al., 2011; Rivero et al., 2015). Tomato plants of similar size were grown under unlimited water supply and exposed to a subsequent drying episode. We asked whether AMF can affect plant water availability (plant available water content), extractability (substrate water potential) and water transport through the substrate (hydraulic conductivity) in equally rooted substrates. To elucidate the physiological relevance of substrate property alterations, we integrated root morphology and substrate hydraulic properties by adopting a root water uptake model. We investigated whether AMF induce changes in the critical substrate water potential that limits plant transpiration under different atmospheric demands.

## Materials and methods

### Plant growth, inoculation, and experimental design

Two weeks after germination in wet sand in the greenhouse, 100 tomato plants (*Solanum lycopersicum* cv. Moneymaker) were transplanted at the three-leaf-stage in 4 L open pots with 3.5 L of a sand/vermiculite mixture (sand: grain size 0.2–1 mm; Euroquarz, Ottendorf-Okrilla, Germany, vermiculite: agra-vermiculite, Pullrhenen, Rhenen, The Netherlands; 1:1 v:v) and set up in the greenhouse in a randomized block design with four blocks. Temperature regulation was set to 22: 17°C (day: night), relative humidity was between 50 and 75% during the day and intensity of photosynthetic active radiation (PAR) at canopy height ranged from 150 to 660 μmol m^−2^s^−1^. Half of the plants were inoculated with *Funneliformis mosseae* BEG12 (MycAgro Laboratory, Breteniere, France) with 10% of the substrate volume. The inoculum carrier material was a mixture of clay and zeolite. Non-mycorrhizal (NM) counterparts were inoculated with a filtrate of the inoculum and the same amount of autoclaved inoculum (2 h, 121°C). The filtrate was produced for every pot by filtration of 200 mL deionized water through Whatman filter (particle retention 4–7 μm; GE Healthcare Europe GmbH, Freiburg, Germany) containing approx. 200 mL of inoculum. The same amount of deionized water (200 mL) was added to mycorrhizal pots. The pots were fertilized approx. every other day the first 3 weeks and subsequently every day with 400 mL of nutrient solution (De Kreij et al., 1997; 40% of full strength) with 10% of the standard phosphate to guarantee good colonization (N: 10.32 mM; P: 0.07 mM, K: 5.5 mM, Mg: 1.2 mM, S: 1.65 mM, Ca: 2.75 mM, Fe: 0.02 mM, pH: 6.2, EC: 1.6 mS). Until the start of the drying cycle 35 days after inoculation and transplanting, ample water conditions were maintained by irrigating with deionized water until pot water capacity and additional water was applied to plates under the pots to guarantee water accessibility throughout whole daytimes. At day 36, all plants were irrigated in the morning to a total of 1,500 mL (≈ pot water capacity), which was sufficient to maintain ample water conditions (WW) during day 36. Afterwards, water was subsequently withheld from pots and two additional harvests were done under water-deficient conditions at day 42 (WD1) and day 47 (WD2).

### Leaf area, root morphology, and fungal colonization

Leaf area (LA) was measured with the LI-3100 Area Meter (LICOR, Lincoln, USA). For leaves that required immediate sampling, leaf length was measured from the first pinnate to the distal end and, area was estimated after Schwarz and Klaering (2001).

Root systems were carefully washed and analyzed with image processing software WinRHIZO Arabidopsis 2012b (Regent instruments, Québec, Canada). Before (further) analysis, washed roots were centrifuged in a common salad spinner to discard adhesive water. The whole root system was divided into three parts (0–5, 5–20, and >20 cm of depth from top), individual parts were weighed and a representative subsample of 25% of fresh matter was analyzed. Weight based upscaling to the bulk root was performed to assess total root length, surface, volume, the mean root diameter and root length density (L_v_) in the substrate volume.

Fungal staining was done with Trypan blue modified after Koske and Gemma (1989). A fine root subsample of 2 g was stored in 15% ethanol, incubated for 20 min at 60°C in 10% KOH, subsequently acidified for 2 min in 2 N HCl and then stained in 0.05% trypan blue in lactic acid for 20 min at 60°C. The percent of mycorrhizal colonization was assessed on 100 root pieces by the grid line intersection method (Giovannetti and Mosse, 1980).

### Substrate hydraulic conductivity and substrate water potential

We used the simplified evaporation method (Schindler, 1980), which is a continuous dry out of a substrate sample under laboratory conditions. Before planting, standard soil sampling cores (*V* = 250 mL, *h* = 5 cm) were introduced into a subset of pots (*n* = 4–6 per treatment and harvest date) in a way that the cylinder diameter covered the central section of the substrate filling level and the depth of the cylinder covered the radius from the center to the rim of the pot. The cylinders were covered with a 2 mm mesh that allowed root and fungal ingrowth and undisturbed harvesting of the incorporated substrate. Roots were cut with a sharp knife along the outside of the mesh while the cylinder remained in the pot. After harvest, sampling cores were weighed and water saturated for 24 h. All measurements were done with the HYPROP system (UMS GmbH, Munich, Germany) according to Peters and Durner (2008). Two tensiometers in different heights (1.25 and 3.75 cm) were introduced into the soil core. The tension was recorded every 10 min and water loss was determined by weighing at least two times a day, resulting in retention functions of the volumetric water content (Θ) at the average tension (h) of both tensiometers which equal the bulk substrate water potential (Ψ_S_). During the measurement water evaporates from the sample surface and the measurement is terminated when air enters the tensiometer ceramic and the tension drops down to 0 hPa. After termination, substrate samples were dried (105°C, 24 h) to obtain the substrate dry mass. For very low water potentials subsamples of the substrate have been taken and measured with C-30 chambers (Wescor Inc., Logan, USA), containing a wet bulb depression psychrometer connected to a PSYRO water potential data logger (Wescor Inc., Logan, USA). After 15 min of temperature equilibration in a water bath (22°C), data were logged every 5 min until occurrence of a plateau. The obtained values were added to the water retention data.

Assuming half of the water flow for evaporation deriving from the upper cylinder height and a linear gradient of volumetric water content (Θ) from bottom to top, a function for the hydraulic conductivity *K*(*h*) can be estimated as:

(1) $$K(h_{i})=\;\frac{0.5q}{\frac{\Delta h}{z_{1}-z_{2}}-1},$$

where *q* is the water flow, Δ*h* is the mean tension difference of the two tensiometers and *z*_*i*_ are the depths of the tensiometers (Peters and Durner, 2008).

Although made for soils, water retention models can also be used for substrate mixes (Fonteno, 1992). Thus, several water retention models were tested (van Genuchten unimodal, van Genuchten bimodal, Brooks and Corey, Ross-Smettem, Fayer-Simmons, Kosugi). Their performance was evaluated based on the Akaike Information Criterion (AICc) for finite sample sizes (Akaike, 1974), which penalizes model complexity, so candidate models with minimum AICc are preferred. To the relationship of Θ vs. Ψ_S_ the bimodal van Genuchten model for water retention was fitted (Durner, 1994) and parameters were estimated with the HYPROP-DES software (UMS GmbH, Munich, Germany). The model allows a mixture of two pore size distributions, which is reasonable for a two component substrate:

(2) $$S_{e}\left(h\right)=\;\sum_{i=1}^{2}\omega_{i}{\left(\frac{1}{1+{\left(\alpha_{i}\left|h\right|\right)}^{n_{i}}}\right)}^{1-\frac{1}{n_{i}}},$$

where *S*_*e*_ is the effective saturation defined as *S*_*e*_ = (Θ - Θ_r_)/ (Θ_S_ - Θ_r_) (Mualem, 1976) with Θ_*r*_ and Θ_*S*_ are the residual and saturated water content, respectively. ω_*i*_ is a weighing factor, *n*_*i*_ is the pore size distribution parameter and α_*i*_ is the reciprocal potential at the air entry water tension of the substrate. The input is the geometric mean of h of both tensiometers (Peters and Durner, 2008). Based on the estimated retention model, the volumetric substrate water content at the time of harvest (Θ_*H*_) was used to compute the substrate water potential at time of harvest (Ψ_*SH*_).

The retention function was coupled to a model for hydraulic conductivities (K) in unsaturated porous media (Mualem, 1976):

(3) $$K\left(h\right)=\;K_{s}S_{e}^{\tau }{\left(\frac{\int_{0}^{S_{e}}h^{-1}\;dS_{e}\left(h\right)}{\int_{0}^{1}h^{-1}dS_{e}\left(h\right)}\right)}^{2},$$

where *K*_*S*_ and τ are the saturated conductivity and a pore tortuosity parameter, respectively. Overall 9 parameters were identified simultaneously from combined retention and conductivity data: Θ_S_, Θ_r_, *w*_2_, α_1_, α_2_, *n*_1_, *n*_2_, *K*_*S*_, and τ. Parameter estimation was carried out as described in Peters and Durner (2008).

### Computation of derived hydraulic parameters

The matrix flux potential (M), which is defined as:

(4) $$M\left(h\right)=\;\int_{h}^{h_{PWP}}K\left(h\right)dh$$

has been shown to be a useful parameter to describe direct soil water limitations as the instantaneous water influx (*T*_*p,s*_) occurring at a maximum soil water potential gradient between the bulk soil (*h*) and the root surface (max(*h*_*rs*_) = *h*_PWP_ = 15,000 cm, M(*h*_PWP_) = 0)

(5) $$\begin{array}{c} T_{p,s}\left(h\right)=\;\rho \left(M\left(h\right)-\;M\left(h_{rs}\right)\right)\;and\\ \;\rho =\;\frac{4z}{r_{0}^{2}-a^{2}r_{m}^{2}+2\left(r_{m}^{2}+r_{0}^{2}\right)\ln\left(\frac{ar_{m}}{r_{0}}\right)} \end{array}$$

of a proxy rhizosphere model geometry (ρ) with the root radius *r*_0_, the mean half inter-root distance *r*_*m*_, the pot height *z* and the relative location of the bulk substrate water potential *a* (0.53) (de Jong van Lier et al., 2008, 2013). Using a prescribed atmospherically demanded potential transpiration (*T*_*p*_,_*a*_), the extent of direct substrate originating water limitations can be expressed as relative transpiration:

(6) $$rT\left(h\right)=\;min\left[\frac{T_{p,s}(h)}{T_{p,a}},1\right].$$

The water potential at the root surface (*h*_rs_) was also calculated from Equation (5), but using the measured plant transpiration rate (*T*_*a*_) at the time of plant harvest in place of *T*_*p,s*_. Adopting the root conductance relation from de Jong van Lier et al. (2013) the resistance of water flow toward roots (R_SOIL_) present at harvest dates was calculated as:

(7) $$R_{SOIL}=\;\frac{2z(h_{r}-h)}{T_{a}r_{m}^{2}\left(ar_{m}/r_{0}\right)},$$

where mean values have been used for bulk substrate water potential (*h*) and actual transpiration *T*_*a*_ to enable treatment comparison at the same soil water content and water flux. Apart from commonly used uniquely sized rhizospheres with a unique radius *r*_*m*_ = (1/πL_V_)^0.5^, we followed a recently proposed approach (Graefe and Bitterlich, submitted), which allows for different rhizosphere sizes *r*_*m,i*_ following the distance distribution of a 2D Poisson point process (Moltchanov, 2012). So, Equation (5) is rewritten as:

(8) $$T_{p,s}\left(h\right)=\;\bar{\rho }(M\left(h\right)-\;M\left(h_{r}\right),$$

where now a mean parameter function ρ is computed over i rhizosphere size classes (Graefe and Bitterlich, submitted).

The volumetric water content Θ in the range between field capacity (FC, pF = 1.8) and the permanent wilting point (PWP, pF = 4.2) is termed plant available water content (PAW), because a proportion of water residing in macro pores would be lost by draining at degrees of saturation that correspond to pF values lower than FC. And, at water contents corresponding to a pF higher than PWP, water resides in micro pores that cannot be extracted by plants (Blume et al., 2009)

### Plant water potentials and evapotranspiration

Water potentials of the compound leaf xylem (Ψ_L_) and the root system (Ψ_R_) were determined using a pressure chamber (Scholander et al., 1965). The first fully expanded leaf was cut at the main stem Both, the leaf and the root system including a short stem base (~5 cm) were covered with plastic foil and immediately inserted into a SKPM 1400 pressure chamber (UP GmbH, Cottbus, Germany). Roots were obtained carefully from pots, separated from adhering substrate while avoiding root tissue damage.

Pressure was increased at a rate of 0.1 bars s^−1^ until the meniscus of xylem sap appeared at the cut surface. For measurements of the leaf lamina potential, leaf discs (2 cm in diameter) have been put into a C-52 sample chamber (Wescor Inc., Logan, USA) containing a wet bulb depression psychrometer connected to a PSYRO water potential data logger (Wescor Inc., Logan, USA). After 15 min of temperature equilibration in a polystyrol box, data were logged every 5 min until occurrence of a plateau. The reference temperature was 22°C. The same part of the lamina was then squashed to destroy cells to determine the osmotic potential accordingly. Turgor pressure (Ψ_T_) was calculated as the difference between the leaf lamina potential and the osmotic potential of the leaf disc.

Plant evapotranspiration was analyzed by weighing of pots every hour during daytime on days of the respective harvests and additionally on day 39 between WW and WD1. For the respective harvest times, larger sets of plants (*n* ≥ 15) were used to estimate actual transpiration rates by fitting a third order polynomial to the daytime time courses between 9 a.m. and 6 p.m.

### Statistical analysis

Statistical analysis (α = 0.05; normal distribution, homogeneity of variances, ANOVA, *t*-test and regressions) were computed with STATISTICA 12 software (StatSoft, Tulsa, OK, USA). In case of violation of assumptions data sets have been log transformed. The goodness of fit (root mean squared error) and the AICc for model selection was computed with the HYPROP-DES software (UMS GmbH, Munich, Germany).

## Results

### Plant growth and substrate hydraulic properties

In order to relate substrate hydraulic properties to plant and fungal growth in a pot specific manner, Tables 1, 2 display data obtained only from those pots that contained sampling cores for hydraulic assessments. Please refer also to the Supplementary Material, which is in the following referred to as S1 to S5.

**Table 1:** Plant development and biomass allocation of mycorrhizal (AM) and non-mycorrhizal (NM) substrates as present at the three harvests (WW, WD1, WD2) during the drying episode.

**Variable**	**Inoculation**	**Harvest time**			**ANOVA**		
		**WW**	**WD1**	**WD2**	**Harvest**	**Inoculation**	**H × I**
					***F*_(2, 23)_*P***	***F*_(1, 23)_*P***	***F*_(2, 23)_*P***
Plant fresh matter [g]	NM	275.0 ±10.9	312.2 ± 7.8	324.1 ± 4.5	**(15.48) < 0.001**	(0.08) 0.778	(0.18) 0.841
	AM	275.7 ± 6.3	313.1 ± 12.3	316.4 ± 8.1			
		A	B	B			
Leaf dry matter [g]	NM	16.7 ± 0.39	19.9 ± 0.63	20.5 ± 0.30	**(26.22) < 0.001**	(4.04) 0.056	(0.20) 0.820
	AM	15.2 ± 0.70	19.3 ± 0.80	19.5 ± 0.63			
		A	B	B			
Root dry matter [g]	NM	1.54 ± 0.27	1.91 ± 0.13	1.78 ± 0.07	**(5.732) < 0.001**	(0.66) 0.425	(0.65) 0.532
	AM	1.29 ± 0.10	1.82 ± 0.18	1.85 ± 0.09			
		A	B	B			
Leaf area [dm^2^]	NM	40.4 ± 1.90	40.4 ± 2.06	40.8 ± 2.41	(0.572) 0.572	(2.78) 0.109	(0.73) 0.491
	AM	42.1 ± 0.97	45.9 ± 1.89	41.7 ± 1.70			
		A	A	A			
Root area [dm^2^]	NM	43.5 ± 4.03	53.2 ± 1.27	51.1 ± 1.61	(10.03) < 0.001	(0.29) 0.597	(0.20) 0.823
	AM	43.0 ± 1.57	54.7 ± 3.71	53.4 ± 1.35			
		A	B	B			
Root/shoot ratio	NM	0.063 ± 0.01	0.060 ± 0.01	0.051 ± 0.01	(2.010) 0.157	(0.13) 0.724	(1.35) 0.280
	AM	0.057 ± 0.01	0.063 ± 0.01	0.058 ± 0.01			
		A	A	A			
Root/leaf area ratio	NM	1.10 ± 0.15	1.32 ± 0.10	1.28 ± 0.10	(3.327) 0.054	(0.61) 0.443	(0.30) 0.742
	AM	1.02 ± 0.02	1.20 ± 0.10	1.30 ± 0.08			
		A	A	A			

*The three harvests occurred 36 days after inoculation under ample water conditions (WW) and 42 and 47 days under water deficient conditions (WD1 and WD2, respectively) after withholding water. The data (mean ± SE, n = 4–6) was analyzed by two way ANOVA (α = 0.05) with significant P-values highlighted in bold. Different capital letters indicate significant differences between harvest dates and asterisks (second column) indicate whether inoculation caused a significant effect (Tukey HSD)*.

**Table 2:** Root development of mycorrhizal (AM) and non-mycorrhizal (NM) substrates as present at the three harvests (WW, WD1, WD2) during the drying episode.

**Variable**	**Inoculation**	**Harvest time**			**ANOVA**		
		**WW**	**WD1**	**WD2**	**Harvest**	**Inoculation**	**H × I**
					***F*_(2, 23)_*P***	***F*_(1, 23)_ P**	***F*_(2, 23)_*P***
Root length density [cm cm^−^^3^]	NM	9.11 ± 0.94	12.01 ± 0.38	12.26 ± 0.34	**(16.15) < 0.001**	(1.51) 0.232	(0.08) 0.920
	AM	9.77 ± 0.36	12.37 ± 0.91	13.12 ± 0.50			
		A	B	B			
Root surface density [cm^2^ cm^−^^3^]	NM	1.24 ± 0.12	1.54 ± 0.04	1.57 ± 0.05	**(15.82) < 0.001**	(0.31) 0.583	(0.21) 0.812
	AM	1.22 ± 0.05	1.59 ± 0.11	1.64 ± 0.04			
		A	B	B			
Root volume density [mm^3^ cm^−^^3^]	NM	53.9 ± 4.15	63.9 ± 0.99	63.7 ± 2.53	**(14.14) < 0.001**	(0.01) 0.917	(0.91) 0.415
	AM	49.4 ± 1.79	65.2 ± 4.23	66.3 ± 1.06			
		A	B	B			
Root diameter [mm]	NM	0.44 ± 0.01 b	0.41 ± 0.01 ab	0.41 ± 0.01 b	**(3.720) 0.040**	**(9.11) 0.006**	**(5.21) 0.014**
	AM	0.40 ± 0.01 a	0.41 ± 0.01 b	0.40 ± 0.01 b			

*The three harvests occurred 36 days after inoculation under ample water conditions (WW) and 42 and 47 days under water deficient conditions (WD1 and WD2, respectively) after withholding water. The data (mean ± SE, n = 4–6) was analyzed by two way ANOVA (α = 0.05) with significant P-values highlighted in bold. In case of significant interaction, values followed by the same small letter are not significantly different (Tukey HSD). Different capital letters indicate significant differences between harvest dates and asterisks (second column) indicate whether inoculation caused a significant effect (Tukey HSD)*.

NM roots did not contain fungal structures. The mean AMF root colonization intensity was 16.4, 25.9, and 30.5% at WW, WD1, and WD2, respectively (see also **Figure 4**). We found intraradical hyphae and arbuscules in all cases, indicating the establishment of a functional symbiosis (not shown).

Plants did not entirely stop growing during the drying episode as root and leaf biomass increased with harvest dates (Table 1). Leaf area did not change during that time, likely resulting from a shrinking lamina due to leaf desiccation, because leaves stayed vital during that period. Thus, root/shoot dry weight ratios were not different between harvest dates, but the root/leaf area ratio slightly increased (*P* = 0.056; Table 1). Roots were growing by approx. 2.5 cm per cm^3^ substrate volume from WW to WD1, but nearly entirely stopped growing between WD1 and WD2 (Table 2). Root surface and volume densities developed accordingly. With exception of slight mycorrhizal effects on root diameters, none of the plant growth parameters was influenced by inoculation with *F. mosseae* and no interaction between the factors harvest date and inoculation was detected. Root volumes constituted 5.16, 6.45, and 6.49% of the substrate volume at WW, WD1 and WD2, respectively. The marginal differences between mycorrhizal and NM pots in root volume densities did not exceed 0.45% of the substrate volume. The coarsely textured substrate had a low bulk density and high porosity (Table 3). During the drying phase total dry porosity declined and a loss of water volume that can be withheld during saturating of the substrate sample, i.e., the saturated water content (Θ_SAT_; Table 3) was observed. Importantly, those soil parameters were not affected by AMF.

**Table 3:** Substrate properties of mycorrhizal (AM) and non-mycorrhizal (NM) substrates as present at the three harvests (WW, WD1, WD2) during the drying episode.

**Variable**	**Inoculation**	**Harvest time**			**ANOVA**		
		**WW**	**WD1**	**WD2**	**Harvest**	**Inoculation**	**H × I**
					***F*_(2, 23)_*P***	***F*_(1, 23)_*P***	***F*_(2, 23)_*P***
Bulk density [g cm^−^^3^]	NM	0.88 ± 0.02	0.92 ± 0.05	0.98 ± 0.01	**(7.01) 0.004**	(0.28) 0.604	(0.64) 0.537
	AM	0.91 ± 0.02	0.92 ± 0.01	0.97 ± 0.02			
		A	AB	B			
Total porosity [–]	NM	0.67 ± 0.01	0.65 ± 0.02	0.63 ± 0.01	**(5.87) 0.009**	(0.40) 0.534	(0.47) 0.632
	AM	0.65 ± 0.01	0.65 ± 0.01	0.64 ± 0.01			
		A	AB	B			
Θ_SAT_ [cm^3^ cm^−^^3^]	NM	0.56 ± 0.12	0.51 ± 0.05	0.51 ± 0.08	**(16.1) < 0.001**	(1.24) 0.277	(0.35) 0.710
	AM	0.57 ± 0.07	0.53 ± 0.09	0.51 ± 0.09			
		A	B	B			

*Θ_SAT_ denotes the saturated water content. The three harvests occurred 36 days after inoculation under ample water conditions (WW) and 42 and 47 days under water deficient conditions (WD1 and WD2, respectively) after withholding water. The data (mean ± SE, N = 4–6) was analyzed by two way ANOVA (α = 0.05) with significant P-values highlighted in bold. Different capital letters indicate significant differences between harvest dates and asterisks (second column) indicate whether inoculation caused a significant effect (Tukey HSD)*.

**Figure 1:**
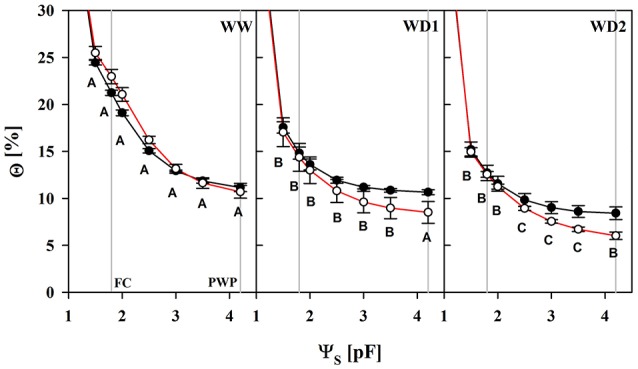
The substrate volumetric water content (Θ) as a function of the substrate water potential (Ψ_S_) of mycorrhizal (white) and non-mycorrhizal (black) substrates sampled at the three harvests. The three harvests occurred 36 days after inoculation under ample water conditions (WW) and 42 and 47 days under water deficient conditions (WD1 and WD2, respectively) after withholding water during the drying episode. Significant differences between harvest dates (*p* < 0.05) were detected from Ψ_S_ = 1.5–4.2. Different capital letters indicate significant differences between harvest dates at particular levels of Ψ_S_. Signifcant differences between mycorrhizal and non-mycorrhizal plants (*p* < 0.05) were detected at Ψ_S_ = 3.5 and 4.2. No significant interaction was detected (mean ± *SE*, *N* = 4–6, two way ANOVA, Tukey HSD). For absolute values and statistical analyses see also Tables S1, S2.

Losses of total porosity from WW to WD2 may partly offset the water retention curves toward lower water contents (Figure 1; Table S1). The substrate lost a proportion of its water capacity when sampled during the drying phase (WD1 and WD2). We fitted the water retention model to every individual dataset, resulting in replicates of water content values at particular reference water potentials. The water content at the so called “field capacity” (FC, pF = 1.8) declined from 22 to 14.6% and 12.8% in average at WW, WD1, and WD2, respectively and was only marginally affected by the mycorrhizal treatment (see Table S1). In contrast, mycorrhizal inoculation caused a change in water contents that correspond to a particular level of water potential in the plant relevant range from FC to the PWP. When harvested after withholding water (WD1 and WD2), Θ comparatively starts to decline at pF = 2.5 in colonized substrates and was significantly lower from pF = 3.5 (see also Table S1). Consistently in all three harvests, the pF in colonized substrates declined less per unit of Θ (see Table S2) as soon as pF = 2 was approached.

**Figure 2:**
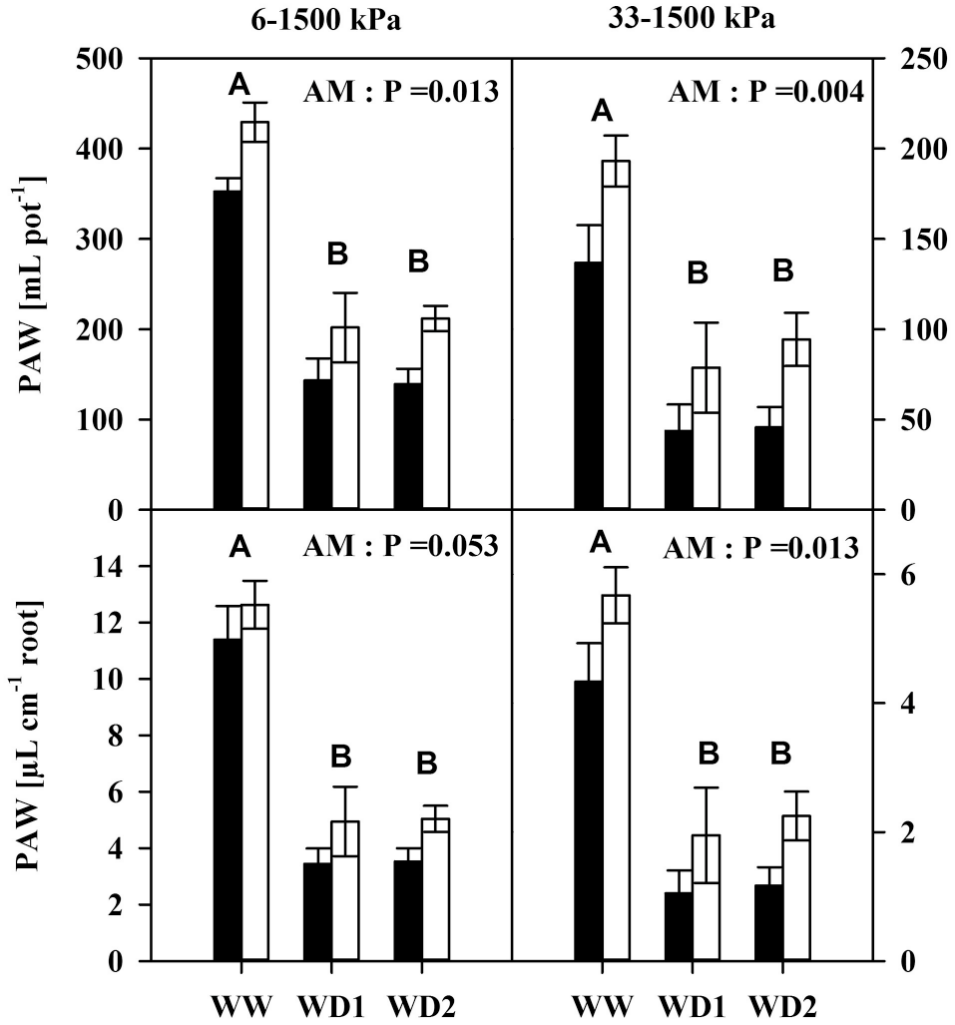
The Plant available water content (PAW) of mycorrhizal (white bars) and non-mycorrhizal (black bars) in substrates **(Top)** and per unit root length **(Bottom)** as present during the three harvests. PAW was quantified as the difference in water content between 6 kPa (pF = 1.8) and 1500 kPa (pF = 4.2) for moist conditions (left), and between 33 kPa (pF = 2.5) and 1,500 kPa for dry conditions (right). The three harvests occurred 36 days after inoculation under ample water conditions (WW) and 42 and 47 days under water deficient conditions (WD1 and WD2, respectively) after withholding water during the drying episode. Significance of factors harvest date (H), inoculation with *F. mosseae* (AM) and their interaction (H × I) was analyzed by two way ANOVA (mean ± *SE, N* = 4–6; α = 0.05). No significant interaction was detected. Different capital letters indicate significant differences between harvest dates (Tukey HSD) and the *P*-value for main factor inoculation (AM) is shown. For complete ANOVA results please refer to the Table S3.

Interestingly, mycorrhization enhanced plant available water contents under moist (pF = 1.8–4.2) and dry conditions (pF = 2.5–4.2) up to 75 and 56 mL, respectively (Figure 2). This was fairly conserved when corrected for the root length (Figure 2, Table S3).

**Figure 3:**
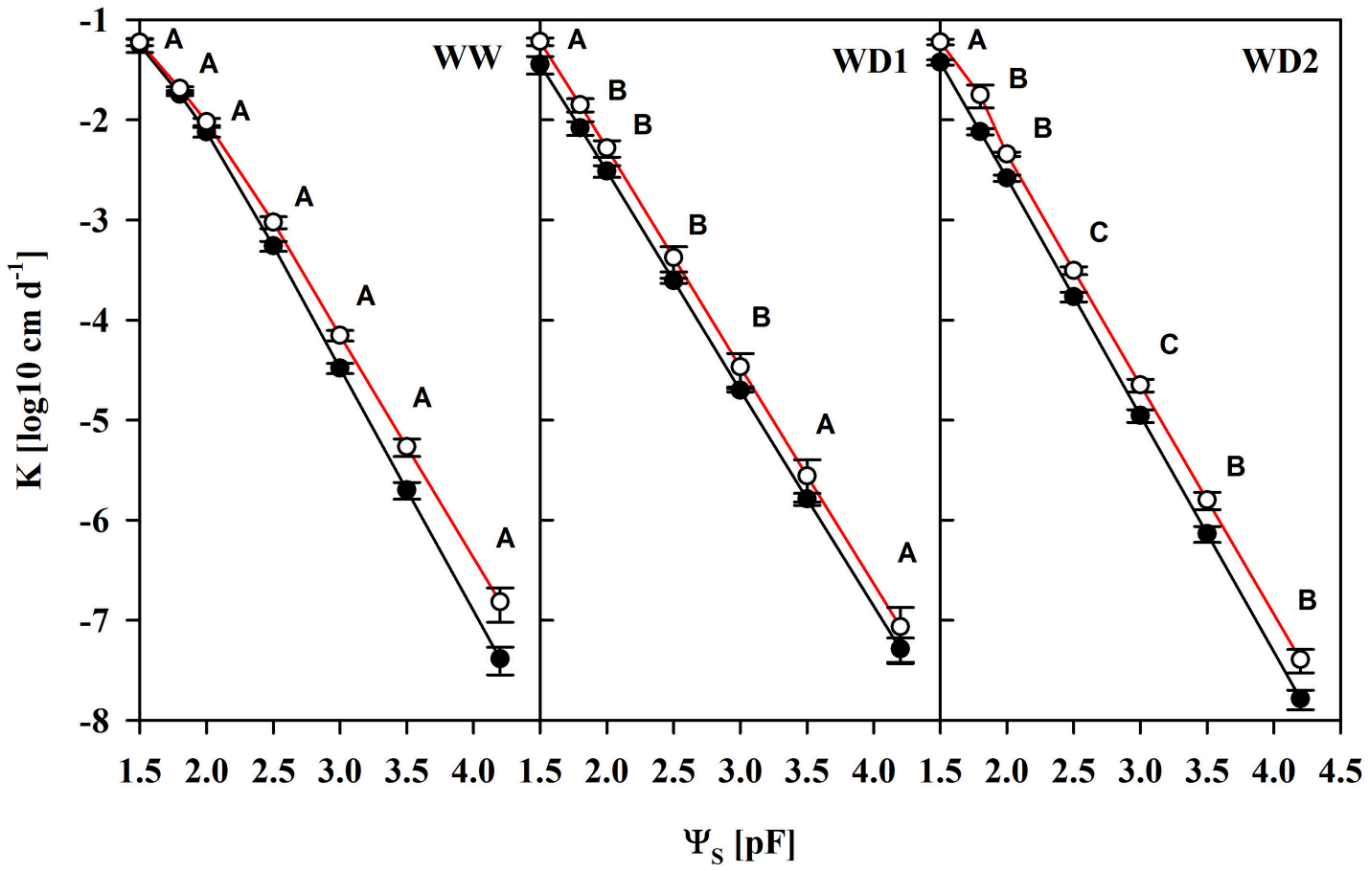
The unsaturated substrate hydraulic conductivity (K) as a function of the substrate water potential (Ψ_S_) of mycorrhizal (white) and non-mycorrhizal (black) substrates sampled at three harvests. The three harvests occurred 36 days after inoculation under ample water conditions (WW) and 42 and 47 days under water deficient conditions (WD1 and WD2, respectively) after withholding water during the drying episode. Significant differences between harvesrfddds a t dates (*p* < 0.05) were detected from Ψ_S_ = 1.8–4.2. Different capital letters indicate significant differences between harvest dates at particular levels of Ψ_S_. Signifcant differences between mycorrhizal and non-mycorrhizal plants (*p* < 0.05) were detected at Ψ_S_ = 1.5–4.2. No significant interaction was detected (mean ± *SE, N* = 4–6, two way ANOVA, Tukey HSD). For absolute values and statistical analyses see also Table S4.

**Figure 4:**
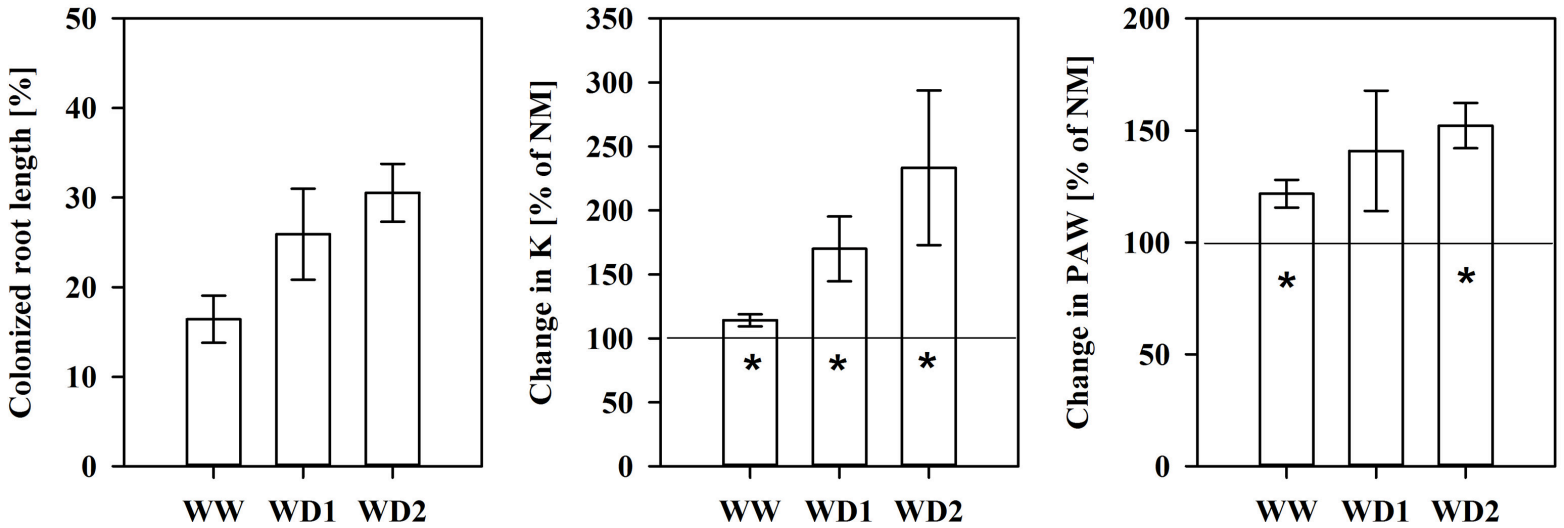
The colonized root length **(Left)**, the relative mycorrhizal improvement in the mean substrate hydraulic conductivity **(Middle)** and plant available water content (PAW, **Right**) between field capacity and the permanent wilting point calculated using the mean of non-mycorrhizal plants as the reference (baseline at 100%) for the harvests during the drying episode. The three harvests occurred 36 days after inoculation under ample water conditions (WW) and 42 and 47 days under water deficient conditions (WD1 and WD2, respectively) after withholding water during the drying episode (mean ± *SE*, *N* = 4–6). Asterisks indicate whether the mean of mycorrhizal plants is higher than that that of non-mycorrhizal plant (*t*-test, α = 0.05).

Another important characteristic of substrates is the water transport capacity through the pore space, i.e., the hydraulic conductivity (K). We found K invariably increased in mycorrhizal substrates at all three harvest dates between pF of 1.8 and 4.2, but also under water saturation (Figure 3, Table S4). K is shown on a logarithmic scale. At WD2 the actual enhancement of average K in mycorrhizal substrates in the FC - PWP moisture range was up to 300% (Figure 4). Absolute values of Θ(pF) and K(pF) declined with harvest time, once substrates started to desiccate. Therefore, we calculated mycorrhizal response ratios with the mean observed in NM pots as the basis. And, AMF stimulation of K from FC to PWP and plant available water contents responded similarly to root colonization intensities observed at the respective harvest dates (Figure 4).

### Plant physiological responses and substrate depletion during drying

Substrates that contained *F. mosseae* were characterized by higher plant available water contents and a lower resistance to water flow (inverse of conductivity) in the plant relevant range of substrate moisture potentials, therefore enhancing the potential supply of water. The water flow toward the root system as driven by plant transpiration will depend on these substrate hydraulic properties. Vice versa, plants are able to sense substrate moisture stress and can quickly adjust transpiration to avoid or delay wilting without direct or relaxed hydraulic feedbacks. To study that, we determined substrate water contents and water potentials present at the time of harvest (Θ_H_, Ψ_SH_).

From WW to WD2 a stronger decline in substrate water contents at harvest was observed in mycorrhizal pots. Both NM and mycorrhizal pots approached plant unavailable water contents (Θ_PWP_) at the last harvest (Table 4). Consistently, substrate water potentials were higher (in means of pF) in mycorrhizal pots at WD1, concurring with higher cumulative evapotranspiration rates. Since the amount of water residing in plants was not altered by AMF inoculation (Table 4), more water was flowing through the mycorrhizal substrate-plant-air continuum between the first two harvests. Cumulative evapotranspiration was about 85 mL higher in colonized pots in the end, which is in good agreement with the improvement in PAW in the substrate observed at WD2 (≈72 mL, see Figure 2). Whole plant transpiration rates were calculated as the average weight loss from harvest to harvest subtracted by the average biomass increment. In mycorrhizal pots, whole plant transpiration rates were declining less between WW and WD1 and stronger between WD1 and WD2. Based on the findings in water retention and the observation made directly at harvest date, the significant interaction in evapotranspiration rates is consistent. From WW to WD1 transpiration depleted the substrate to a degree of saturation where the stress (pF) in mycorrhizal pots declined less per unit water content and substrate conductivity was higher. Remarkably, leaf turgidity, the leaf xylem water potential and the root water potential were equal in NM and mycorrhizal pots, although mycorrhizal plants grew in stronger water depleted substrates (Table 4). At WD2 leaves of two NM and two mycorrhizal plants lost turgidity. Hence, NM and mycorrhizal plants were equally on the brink of wilting at WD2 and the mean negative turgidity of colonized plants at WD2 is not indicating a general turgor loss. Within the rhizosphere, the resistance of water flow toward roots (R_SOIL_) and the water potential estimated at the root surface (Ψ_RS_) was conserved in colonized pots, although mycorrhizal substrates were more desiccated (Table 4).

**Table 4:** Substrate and plant hydraulic properties observed at harvest of mycorrhizal (AM) and non-mycorrhizal (NM) substrates as present at the three harvests (WW, WD1, WD2) during the drying episode.

**Variable**	**Inoculation**	**Harvest time**			**ANOVA**		
		**WW**	**WD1**	**WD2**	**Harvest**	**Inoculation**	**H × I**
					***F*_(2, 23)_*P***	***F*_(1, 23)_*P***	***F*_(2, 23)_*P***
Θ_H_ [%]	NM	47.7 ± 0.5	12.7 ± 1.4	8.5 ± 0.9	**(795) <0.001**	**(4.77) 0.039**	(0.20) 0.824
	AM^*^	46.5 ± 0.9	10.8 ± 1.9	6.3 ± 0.3			
		A	B	C			
Θ_PWP_ [%]	NM	11.2 ± 0.43	10.9 ± 0.3	8.4 ± 0.7	**(14.8) <0.001**	**(7.62) 0.011**	(1.06) 0.364
	AM^*^	10.7 ± 0.69	9.0 ± 1.1	6.0 ± 0.4			
		A	A	B			
Ψ_SH_ [pF]	NM	1.00 ± 0.02	2.60 ± 0.24	3.48 ± 0.12	**(88.5) <0.001**	**(4.72) 0.041**	(1.15) 0.332
	AM^*^	1.01 ± 0.01	3.19 ± 0.45	3.81 ± 0.03			
		A	B	C			
Ψ_RS_ [pF]	NM	1.01 ± 0.01	3.56 ± 0.01	3.74 ± 0.02	**(3,409) <0.001**	(1.20) 0.283	(1.50) 0.250
	AM	1.01 ± 0.01	3.57 ± 0.01	3.71 ± 0.01			
		A	B	C			
K_H_ [log10 cm d^−1^]	NM	0.28 ± 0.21	−3.79 ± 0.56	−6.12 ± 0.31	**(94.0) <0.001**	(2.84) 0.106	(1.03) 0.372
	AM	0.37 ± 0.06	−5.03 ± 1.08	−6.56 ± 0.13			
		A	B	C			
R_SOIL_ [d cm^−1^]	NM	4^*^10^−1^	2.2^*^10^4^	2.7^*^10^5^	**(1070) <0.001**	(1.15) 0.294	(2.45) 0.109
	AM	4^*^10^−1^	2.7^*^10^4^	7.7^*^10^4^			
		A	B	C			
Cumulative evapotranspiration [mL]	NM	222.8 ± 6.2 a	1187 ± 25.0 b	1704 ± 32.6 d	**(4,438) <0.001**	**(6.93) 0.015**	**(7.25) 0.002**
	AM^*^	222.1 ± 4.2 a	1277 ± 19.6 c	1790 ± 30.1 d			
Evapotranspiration rate [mL d^−1^]	NM	247.8 ± 6.0 d	205.5 ± 4.3 b	122.9 ± 3.3 a	**(420) <0.001**	(1.10) 0.305	**(2.36) 0.045**
	AM	246.2 ± 4.4 d	220.7 ± 3.4 c	122.4 ± 3.8 a			
Water in the plant [g]	NM	249.1 ± 9.9	278.7 ± 8.30	287.2 ± 4.9	**(10.8) <0.001**	(0.10) 0.922	(0.18) 0.838
	AM	252.0 ± 5.7	282.3 ± 10.8	282.6 ± 7.5			
		A	B	B			
Turgor pressure [MPa]	NM	0.38 ± 0.05	0.35 ± 0.08	0.14 ± 0.19	(3.28) 0.056	(0.16) 0.692	(0.19) 0.828
	AM	0.35 ± 0.03	0.38 ± 0.09	−0.01 ± 0.15			
		A	A	A			
Leaf water potential [MPa]	NM	−0.69 ± 0.05	−0.80 ± 0.09	−0.98 ± 0.03	**(13.2) <0.001**	(0.01) 0.967	(0.44) 0.648
	AM	−0.51 ± 0.05	−0.84 ± 0.12	−1.01 ± 0.05			
		A	AB	B			
Root water potential [MPa]	NM	−0.06 ± 0.01	−0.55 ± 0.07	−0.65 ± 0.04	**(68.9) <0.001**	(2.21) 0.151	(1.19) 0.321
	AM	−0.06 ± 0.01	−0.51 ± 0.06	−0.51 ± 0.06			
		A	B	B			

*Θ_H_, Ψ_SH_, and K_H_ denote the substrate water content, water potential and substrate hydraulic conductivity present at harvest, respectively. Θ_4.2_ denotes the water content at the permanent wilting point. Ψ_RS_ and R_SOIL_ are the mean water potential estimated at the root surface and the resistance of water flow to roots, respectively. The three harvests occurred 36 days after inoculation under ample water conditions (WW) and 42 and 47 days under water deficient conditions (WD1 and WD2, respectively) after withholding water. The data (mean ± SE, n = 4–6, transpiration data: n ≥15) was analyzed by two way ANOVA (α = 0.05) with significant P-values highlighted in bold. In case of significant interaction, values followed by the same small letter are not significantly different (Tukey HSD). Different capital letters indicate significant differences between harvest dates and asterisks (second column) indicate whether inoculation caused a significant effect (Tukey HSD)*.

### Limitation of transpiration by restriction of substrate water flow

Measured transpiration rates under ample water conditions (WW) have been highest at noon (1.6 cm d^−1^) and lowest in the morning and evening (0.4 cm d^−1^). Mean daytime transpiration rates have been 1.3, 0.9, and 0.6 cm d^−1^ at WW, WD1 and WD2, respectively. The atmospheric conditions during the experiment constituted low to moderate atmospheric demands (*T* = 22: 17°C, day: night; rH = 50–75%; PAR = 150–660 μmol m^−2^s^−1^). Based on our measurements, we chose actual transpiration rates of 2 cm d^−1^ for high atmospheric demands, which would be realistic at higher temperatures, higher light intensities and/or lower rH. For low and moderate atmospheric demands, 0.5 and 1 cm d^−1^ were chosen as scenarios that would apply for growing conditions present in the early and late morning/afternoon, respectively, or under drought.

**Figure 5:**
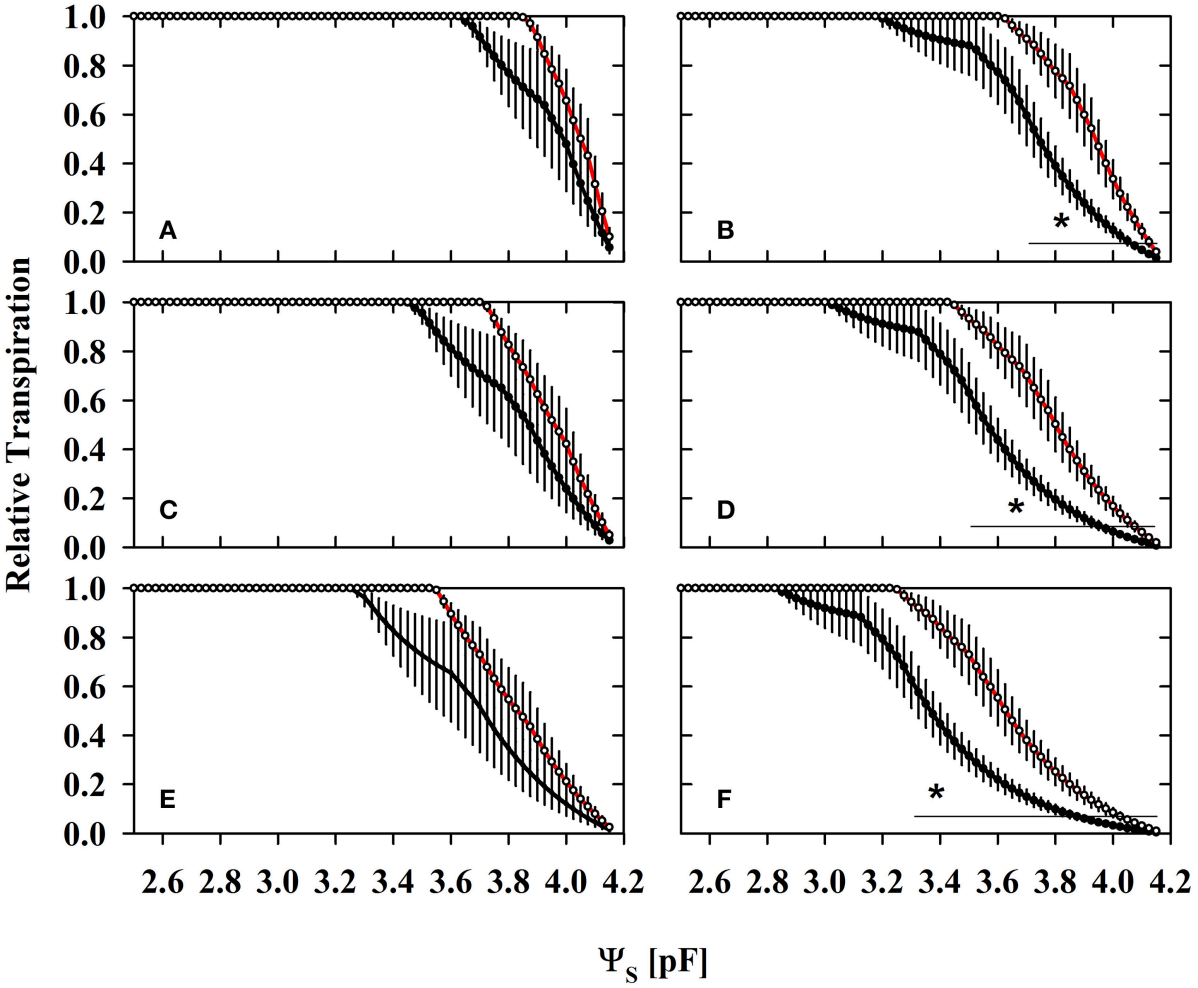
The substrate related water limitation expressed as relative transpiration (potential root water influx/transpiration demand) under different levels of atmospheric demands **(A,B)** 0.5 cm d^−1^; **(C,D)** 1 cm d^−1^; **(E,F)** 2 cm d^−1^ in mycorrhizal (red lines) and non-mycorrhizal (black lines) substrates harvested under ample water conditions (WW; **A,C,E)** and under water deficient conditions (WD2; **B,D,F)** (mean ± SE, *N* = 4–6). The highest measured actual transpiration rates during the experiment have been 1.6 cm d^−1^ at noon and lowest in the morning and evening (0.4 cm d^−1^) at WW. Average daytime transpiration rates have been 1.3, 0.9, and 0.6 cm d^−1^ at WW, WD1, and WD2, respectively under growing conditions with moderate atmospheric demands (*T* = 22:17°C, day: night; rH = 50–75%; PAR = 150–660 μmol m^−2^s^−1^). A value of 1 denotes that water flux allowed by substrates is higher than the level of assumed actual transpiration rates (0.5, 1, and 2 cm d^−1^). Values lower than one indicate substrate water flux limitations, i.e., substrate water flow rates are smaller than actual transpiration rates. Lines with an asterisk denote the ra006Ege where relative transpiration was different between NM and AM pots (Students *t*-test: α = 0.05).

Figure 5 illustrates the limitation of transpiration by substrate water flux expressed as relative transpiration rates (potential transpiration allowed by substrates/actual transpiration demands). As long as relative transpiration is equal to 1, the potential substrate water flux is higher than the actual plant transpiration demand and not limiting. Relative transpiration lower than 1, indicates transpiration limitation by substrate water flux.

**Figure 6:**
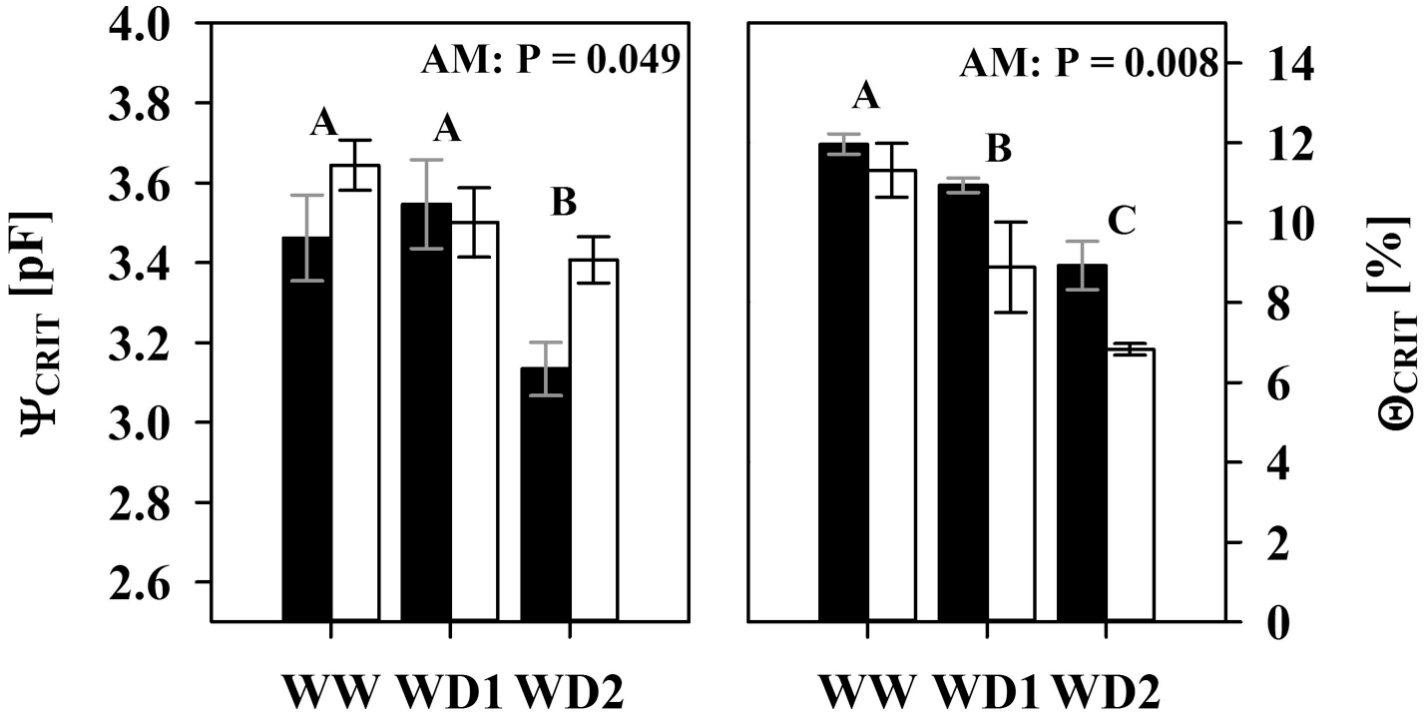
The critical substrate water potential (Ψ_CRIT_) and water content (Θ_CRIT_) that start to limit root water influx under high atmospheric demands (2 cm d^−1^) of mycorrhizal (white bars), and non-mycorrhizal (black bars) plants as sampled during the three harvests. The three harvests occurred 36 days after inoculation under ample water conditions (WW) and 42 and 47 days under water deficient conditions (WD1 and WD2, respectively) after withholding water during the drying episode. Significance of factors harvest date (H), inoculation with *F. mosseae* (I) and their interaction (H × I) was analyzed by two way ANOVA (mean ± *SE, N* = 4–6, α = 0.05). No significant interaction was detected. Different captial letters indicate significant differences between harvest dates (Tukey HSD) and the *P*-value for main factor inoculation (AM) is shown. For complete ANOVA results please refer to the Table S5.

Relative transpiration already decreased at a lower pF in NM substrates than in colonized substrates (Figure 5). The AMF effect at WW (Figures 5A,C,E) is not yet significant, but becomes clearly pronounced at the last harvest where AMF root colonization was highest (WD2; Figures 5B,D,F). Similar to WW, no significant differences were observed at WD1 (not shown). The critical drought intensity (pF) at which transpiration becomes limited is highest under low transpirative demands (Figures 5A,B) and gradually shifts to lower drought intensities at moderate (Figures 5C,D) and high transpirative demands (Figures 5E,F). When colonized by AMF, substrates harvested at WD2 can provide water at sufficient rates to match high transpirative demands (Figure 5F) until a pF of 3.24 is reached. In NM pots, substrate limitation already set in at a pF of 2.83 (Figure 5F). Expressed on an absolute basis, under those conditions, mycorrhizal pots are able to fulfill high transpiration demands for an extra substrate water potential depletion of 1,079 hPa. The critical water content (Θ_CRIT_) where transpiration becomes limited by substrate drought was reduced in mycorrhizal substrates at all three harvest dates and the mycorrhizal effect becomes more pronounced with experiment duration (Figure 6; Table S5). The critical soil water potential (Ψ_CRIT_) was also reduced except at WD1 (Figure 6; Table S5), which is coinciding with the smallest differences in hydraulic conductivity between pF 3 and pF 4.2 (see Table S4).

## Discussion

As anticipated, we did not observe a growth response to AMF inoculation, but intraradical hyphae, arbuscules and the maintenance of the plant hydraulic state in stronger water depleted substrates indicate a symbiotic relationship, because the latter is a common observation in mycorrhizal experiments. An extensive review (including more than 200 studies, 90 host species and at least 22 AMF species) revealed that in 75% of the cases where soil moisture was measured, mycorrhizal plants were observed to deplete the soil water more thoroughly, before achieving a similar shoot response, which is often not associated with better growth (Augé, 2001). This was verified here. We used a substrate for our study that is reproducible and characterized by a high porosity and a low bulk density. Similar to other horticultural substrates this allows easy extraction of water with a low risk of inducing hypoxia under high saturation (Fonteno, 1992). We are aware that the applied water retention model was originally developed for soils. However, such models can be used also for horticultural substrates (Fonteno, 1992), especially when they fit well to measured data as judged by the AICc.

The loss of substrate water capacity with experiment duration could be related to several factors, but the root volume increment alone is not large enough to explain the changes in Θ_SAT_. There was no substrate shrinkage detected, when substrates desiccated during the water retention measurements. The time of re-saturation could have been too short (24 h), because substrates that contain vermiculite may require longer times to fully saturate than soils (Fonteno, 1992). Increased water repellency could be a factor. Substrate water repellency can increase with root and fungal colonization, and with drought, but longer water contact times can recover wettability (Doerr et al., 2000). Although not predicted, this also constitutes the realistic scenario for the plant during the drying episode and would also apply when pots would be re-irrigated. We found the smallest differences in hydraulic properties at WD1, which requires further research. Substrates containing AMF have been stronger depleted of water in the course of the experiment, which putatively increases repellency and may have partly offset positive mycorrhizal effects caused by changes in pore distribution or connectivity.

The influence of AMF on water retention is not abundantly reported and especially scarce under experimental conditions, where rooting is equal like here or in Augé et al. (2001). Our finding of a stronger decline in water contents per unit pF in mycorrhizal substrates is consistent with observations made Augé et al. (2001) in an equally rooted mix of loamy soil and quartz sand. Both studies have in common that water retention curves are characteristic for a coarsely textured substrate with a significant proportion of sand. On such coarsely textured substrates, differences in substrate structure and water retention induced by AMF would be expected to be strongest (Leifheit et al., 2014; Querejeta, 2017).

Soil water retention is largely determined by texture and soil structure while roots and AMF can affect structure. Although roots may have the largest influence on structure (Hallett et al., 2009; Daynes et al., 2013), hyphae are sufficient to influence substrate properties like water repellency (Rillig et al., 2010) and can promote the formation of aggregates within rooted substrates (Augé et al., 2001; Leifheit et al., 2014). A hierarchical development of aggregates from micro-aggregates (<20 μm) is probably not very pronounced in our case, because the sand and the vermiculite grain size was larger than 200 μm and the experiment duration was rather short. Although vermiculite particles may also partly disintegrate with rooting or drying and the carrier material also provided some clay particles, substrate water retention characteristics were typical for a coarse texture. On dune sand or sandy soils, sand particles are enmeshed and entangled to aggregates by hyphae and adhere to the hyphal surface (Clough and Sutton, 1978; Forster, 1979) that is covered by mucilage and polysaccharides among other “sticky” substances (Miller and Jastrow, 2000; Rillig and Mummey, 2006). Such a direct effect of hyphae is likely to occur on the used substrate, because its coarse texture requires hyphae or roots bridging large pores (Miller and Jastrow, 2000) and probably does need shorter colonization times than a hierarchical formation of aggregates from e.g., microbial and plant debris. Those processes would influence the size, shape and connectivity of the pore space where the water flow through substrates occurs.

We did not find any changes in total porosity upon AMF colonization. The effects of AMF on total dry porosity might just be marginal, either because the volume of hyphae and spores is too small to detect a change in pore volume and/or AMF induces processes that create and reduce pore volume simultaneously and thus compensate each other. Significant changes in substrate water retention indicate reorganization in discrete pore size distributions (Daynes et al., 2013). Indeed, the AMF induced increases in PAW without changes in total porosity found here is consistent with another study using a substrate deriving from coarse spoil (Daynes et al., 2013) and may be indicative for a gain of partial porosity of the pore space. Because total porosity and the saturated water content were not influenced by AMF, the suggested increase of porosity related to plant extractable pore volume has come by the expense of other pores or pore property transformations. Although porosity inside aggregates might not be strongly altered by AMF (Hallett et al., 2009), aggregate formation might alter inter- and intra-aggregate proportions of total porosity or the interconnectivity of the inter-aggregate pore space. The degree of aggregation increases with hyphal length (Miller and Jastrow, 2000) and we found PAW to relatively increase with time and root colonization as a surrogate for fungal development. Many other processes occur in biologically active substrates that could change the water potential at a particular degree of saturation. Hydrophobicity influences the contact angle of the liquid-solid phase (Letey et al., 1962) and hydrophobicity of hyphae or exudates could change the effective wettable pore space. AMF may also influence the microbial community that alters porosity, water repellency or change the properties of particle surfaces. Future studies could use sampling cores covered by meshes only allowing fungal access on substrates that facilitate harvest of hyphae to further elucidate these mechanisms (Querejeta, 2017).

Hydraulic substrate conductivity measures how water can be transmitted through the pore space and depends on pore connectivity, pore geometry and tortuosity (Durner, 1994). Some studies showed a significant water transport across compartments only connected by extraradical hyphae under ongoing plant transpiration (Khalvati et al., 2005; Ruth et al., 2011). Those observations may be plant driven, because intrahyphal transport of P seems to be accelerated by the plant transpiration stream along intrahyphal water potential gradients (Bitterlich and Franken, 2016; Kikuchi et al., 2016). Hydraulic conductivity as a strict physical substrate property, which does not require plant or fungal activity, was improved upon AMF colonization. We did not find any other study to compare our mycorrhizal effects. By theory, unsaturated hydraulic conductivity is mainly determined by the largest water filled pores and is thus related to pore size distribution (Mualem, 1976; Durner, 1994). However, varying pore geometry as induced by aggregate formation can alter hydraulic conductivity although substrate water retention remains the same (Durner, 1994). We cannot clarify the mechanistic background of our observations in water retention and conductivity apart from fungal presence, but the decreased resistance of water flow may indicate altered pore space geometry. In addition to aggregation processes, hyphae may bridge air filled pores and restore root-substrate contact, fill voids of particular pore sizes or retain pore connectivity by smoothing the surface profile of particles. Non-septate (dead, disrupted) AMF hyphae of about 5 μm in diameter (Staddon et al., 2003) could also constitute less tortuous bio-pores that would be emptied at substrate water potentials considered as low moisture conditions. If the quantity of hyphae or pores left behind after hyphae degradation could explain our observation cannot be answered here, but is at least debatable, because unrealistic high flow rates within hyphae would be required to significantly affect plant water uptake (George et al., 1992). But, roots and AMF hyphae are able to enhance the structural pore volume by several magnitudes higher than can be explained by the biomass volume alone (Milleret et al., 2009).

Changes in hydraulic properties upon AMF inoculation can have consequences for plants to take up water, their stress response and solute transport into the vicinity of roots. To study the impact of AMF on plant activity we performed the quantitative limitation analysis. From water retention and hydraulic conductivity functions alone, it remains elusive, which impact the observed AMF effects have on the plant's ability to acquire water from the substrate. Whole plant transpiration depends on atmospheric conditions and plant size. Plant transpiration will only become restricted by substrate water flux when the water flow to the root surface provided by the whole pot cannot compensate aerial transpirative demands. The substrate water flux will depend on the total amount of water (Θ × substrate volume), water extractability (Ψ_S_), water mobility (K) and the rhizosphere size (rooting density, root diameter). We integrated those factors with the approach described in section 2.4 in order to assess substrate derived limitation to transpiration quantitatively under different atmospheric demands.

For the first time we show here that substrate water flux limitation to plant transpiration is delayed to stronger drought intensities in mycorrhizal substrates. This is caused by effects that relate to the water flow within the rhizosphere outside of roots. Our analysis illustrates that the mass flow of substrate water, and with that, solute transport, can be maintained in mycorrhizal substrates during higher drought intensities under distinct transpirative demands. This may be of high ecological relevance and could contribute to the frequently observed AMF growth promotion and nutrient acquisition in drought stress experiments (Augé, 2001). Moreover, the limiting drought intensity decreased with the transpirative demand induced by the atmosphere. We are convinced that this important to understand how growing conditions, which vary largely between studies, are decisive for intermittent mycorrhizal effects and the choice and effectiveness of distinct drought treatments.

Plants have developed mechanisms to sense substrate drought. The mechanism has a hydraulic and a biochemical component (ABA) and the message serves to inform the plant about the soil water status (Tardieu and Davies, 1993). This enables plants to avoid exhaustive behavior and regulate transpiration via stomatal movement (Tardieu and Simonneau, 1998). From an ecological point of view this is mandatory to sustain viability and prolong survival times under such conditions. Indeed, we found indication for such reactions. Under our experimental conditions, water flux limitations to transpiration would only occur at drought intensities that were achieved at the end of the drying episode (WD2), but plant transpiration (calculated as the daily average between harvests) already decreased in between the first two harvests. This requires a plant feed-forward response to substrate drought. Mycorrhizal plants did transpire more water in the early phase of drying, which suggests a higher leaf conductance on the plant level. Mycorrhizal plants may not show altered sensitivities of stomatal conductance to xylem [ABA] (Duan et al., 1996), but the authors suggested that mycorrhizal plants are able to better scavenge water in drier soils, which alleviates ABA production in roots. Within the early drying phase (WW to WD1) plants already passed the phase where the substrate pF in mycorrhizal pots declined less with a reduction of Θ caused by plant activity (see Table S3). In combination with the improvements in substrate conductivity this could have caused an alleviation of the stress response in mycorrhizal roots and in turn, to higher transpiration.

Actually, for pot cultures, mycorrhizal plants exerted a more exhaustive behavior. However, such behavior of mycorrhizal plants is potentially advantageous for water and nutrient acquisition in scenarios of alternate irrigation as applied in many reductionist pot experiments. Indeed, AM plants have grown better under pulsed irrigation treatments (Birhane et al., 2012). And, in an earlier study we found stomatal condcutance to be improved in mycorrhizal tomatoes, colonized by the same fungus on the same substrate (Boldt et al., 2011). In field scenarios, where water contents decline less quickly due to subsequent water delivery from the periphery (Tardieu and Simonneau, 1998), a longer duration of phases, where stress responses are alleviated in mycorrhizal plants, is possible when those effects also occur in soils.

## Conclusions

Our study revealed that inoculation with arbuscular mycorrhiza can result in an improvement in water availability and water transport within colonized substrates. Physiologically, this indicates that plants may experience or sense less stress at the root surface at equal substrate moisture, when substrate moisture declines. For experimental systems that investigate the drought tolerance of mycorrhizal plants, it would be important to consider those effects when particular irrigation treatments are used. The fact that mycorrhiza delays the critical substrate water potential for transpiration inhibition to stronger stress levels cannot be seen as a general transference of drought tolerance in every scenario. In pots, the latter can lead to higher resource acquisition, because the mass flow in substrates declines later, but would also cause higher resource depletion rates. To benefit from that, a timely irrigation in e.g., hydroponic pot systems would be required. In systems where water flow from the periphery is possible, e.g., in field scenarios, the observed mycorrhizal effect putatively increases plant resource use efficiency, when total availability of water and solutes increases by subsequent delivery from areas outside the ambit of roots. This is either driven by higher transpiration or allowed by higher substrate conductivity. Finally, the suggested increment in substrate mass flow by AMF under severe drought may contribute to the acquisition of mobile nutrients such as NO_3_ and K under drought conditions.

Further studies are required to elucidate whether easier water extractability and improved hydraulic conductivity at equal drought intensity in mycorrhizal substrates would lead to alleviation of the physiological stress response in the plant. But if this is the case, mycorrhizal plants could be able to invest more resources in biomass development instead of e.g., osmotic adjustments when substrate moisture declines before transpiration is limited by substrate moisture. And, they sustain water and nutrient acquisition under severe drought, because of delayed substrate born limitations to transpiration. Such scenarios would have a strong ecological significance and would reason targeting the use of AMF in crop production systems. Therefore, mycorrhizal effects on substrate hydraulic properties are worth investigating on different crops and substrates with different textures.

In the future, the underlying mechanisms responsible for alterations of substrate hydraulic properties by mycorrhizal colonization could be investigated by applying water retention measurements on substrate proportions that exclude root in-growth and/or by the use of mutants resistant to mycorrhizal colonization. Subsequently, with such experimental systems, changes in hydraulic properties could then be associated directly to hyphal length, induction of aggregation, water repellency or pore clogging. Standard methods like sieving techniques, measurements of water contact angle or water drop penetration tests, only to name a few, are well established, but have only scarcely been used in combination with water retention assessments to quantify mycorrhizal effects. This however, is crucial to understand the relevance of direct extraradical mycorrhizal effects for plant physiology and for the induction of plant drought stress response. Furthermore, since water and solute transport are closely linked, our proposed approach can help distinguishing between direct hyphal delivery of N and P and indirect mycorrhizal effects that alleviate substrate mass flow restrictions, by using isotopic labeling techniques.

## Author contributions

MB: conducted the experiments, analyzed the data and wrote the manuscript; MS: set up the HYPROP method, revisedthe manuscript and contributed to writing; JG: conducted the experiments, developed and conducted root water uptake modeling and revised the manuscript.

### Conflict of interest statement

The authors declare that the research was conducted in the absence of any commercial or financial relationships that could be construed as a potential conflict of interest.
