# Supplementary material for: Arbuscular Mycorrhiza Alleviates Restrictions to Substrate Water Flow and Delays Transpiration Limitation to Stronger Drought in Tomato
Source: Front Plant Sci. 2018 Feb 16;9:154. doi: 10.3389/fpls.2018.00154 (PMC5820414; doi:10.3389/fpls.2018.00154)
Supplement: Supplementary file 3 [file Table3.DOCX]

**Supplementary material:**

S 3: The plant available water content (PAW) of mycorrhizal (AM) and non-mycorrhizal (NM) substrates in the wet and dry pF range observed at the three harvests (WW, WD1, WD2) during the drying episode.

| Plant available water | Inoculation | Harvest time | | | ANOVA | | |
| --- | --- | --- | --- | --- | --- | --- | --- |
| PAW (pF-pF) |  | WW | WD1 | WD2 | Harvest | Inoculation | H x I |
|  |  |  |  |  | (F_(2,23)_) P | (F_(1,23)_) P | (F_(2,23)_) P |
| PAW (1.8-4.2)  [mL pot^-1^] | NM*  AM* | 353±14.6  429±21.9  A | 146±28.6  205±39.1  B | 150±18.4  228±15.1  B | **(26.97) <0.001** | **(7.21) 0.013** | (0.075) 0.927 |
| PAW (2.5-4.2)  [mL pot^-1^] | NM*  AM* | 137±20.8  193±14.0  A | 44±17.2  80±25.4  B | 49±12.0  102±15.8  B | **(17.87) <0.001** | **(10.20) 0.004** | (0.157) 0.855 |
| PAW (1.8-4.2)  [mL cm^-1^ root] | NM*  AM* | 11.39±1.20  12.63±0.85  A | 3.51±0.64  5.03±1.25  B | 3.81±0.50  5.43±0.50  B | **(49.46) <0.001** | (4.17) 0.053 | (0.027) 0.973 |
| PAW (2.5-4.2)  [mL cm^-1^ root] | NM*  AM* | 4.33±0.60  5.67±0.43  A | 1.07±0.42  1.98±0.75  B | 1.26±0.31  2.42±0.41  B | **(26.98) <0.001** | **(7.22) 0.013** | (0.075) 0.928 |

The three harvests occurred 36 days after inoculation under ample water conditions (WW) and 42 and 47 days under water deficient conditions (WD1 and WD2, respectively) after withholding water. The data (mean ± SE, n = 4-6) was analyzed by two way ANOVA (α = 0.05) with significant P values highlighted in bold. Different capital letters indicate significant differences between harvest dates and asterisks (second column) indicate whether inoculation caused a significant effect (Tukey HSD).
