# Supplementary material for: Arbuscular Mycorrhiza Alleviates Restrictions to Substrate Water Flow and Delays Transpiration Limitation to Stronger Drought in Tomato
Source: Front Plant Sci. 2018 Feb 16;9:154. doi: 10.3389/fpls.2018.00154 (PMC5820414; doi:10.3389/fpls.2018.00154)
Supplement: Supplementary file 5 [file Table5.DOCX]

**Supplementary material:**

S 5: The critical substrate water potential (Ψ_CRIT_) and water content (Θ_CRIT_) of mycorrhizal (AM) and non-mycorrhizal (NM) substrates at which transpiration becomes quantitatively limited by substrate water flow under high atmospheric demands (2cm d^-1^) and conditions observed at the three harvests (WW, WD1, WD2) during the drying episode.

| Variable | Inoculation | Harvest time | | | ANOVA | | |
| --- | --- | --- | --- | --- | --- | --- | --- |
|  |  | WW | WD1 | WD2 | Harvest | Inoculation | H x I |
|  |  |  |  |  | (F_(2,23)_) P | (F_(1,23)_) P | (F_(2,23)_) P |
| Ψ_CRIT_ [pF] | NM*  AM* | 3.46±0.11  3.64±0.06  A | 3.55±0.11  3.50±0.09  A | 3.13±0.07  3.41±0.06  B | **(8.57) 0.002** | **(4.32) 0.049** | (2.01) 0.156 |
| Θ_CRIT_ [%] | NM*  AM* | 11.96±0.26  11.30±0.67  A | 10.93±0.18  8.88±1.13  B | 8.93±0.61  6.83±0.14  C | **(17.84) <0.001** | **(8.55) 0.008** | (0.758) 0.480 |

The three harvests occurred 36 days after inoculation under ample water conditions (WW) and 42 and 47 days under water deficient conditions (WD1 and WD2, respectively) after withholding water. The data (mean ± SE, n = 4-6) was analyzed by two way ANOVA (α = 0.05) with significant P values highlighted in bold. Different capital letters indicate significant differences between harvest dates and asterisks (second column) indicate whether inoculation caused a significant effect (Tukey HSD).
